# Measurement instruments for fast and frequent data collection during the early phase of COVID-19 in Germany: reflections on the Mannheim Corona Study

**DOI:** 10.1186/s42409-022-00030-5

**Published:** 2022-02-22

**Authors:** Carina Cornesse, Marisabel Gonzalez Ocanto, Marina Fikel, Sabine Friedel, Ulrich Krieger, Tobias Rettig, Annelies G. Blom

**Affiliations:** 1 SFB 884 Political Economy of Reforms, University of Mannheim, Mannheim, Germany; 2 German Socio-Economic Panel (G-SOEP), German Institute for Economic Research (DIW Berlin), Berlin, Germany; 3 Data Center Social Cohesion, Research Institute Social Cohesion (RISC), University of Bremen, Bremen, Germany; 4 DIGSSCORE, Department of Administration and Organization Theory, University of Bergen, Bergen, Norway

**Keywords:** Online panel, COVID-19, Questionnaire, Survey data, Contact tracing app, Anxiety, Attitudes, Pandemic

## Abstract

The outbreak of the COVID-19 pandemic has led to a vast increase in the demand for fast, frequent, and multi-faceted data to study the impact of the pandemic on people’s lives. Existing data collection infrastructures had to be adapted quickly during the early phase of the pandemic to meet this data demand. Our research group contributed to this by conducting the Mannheim Corona Study (MCS), a longitudinal probability-based online survey, in a daily rotating panel design that took place from March 20 through July 10, 2020. The fast-and-frequent panel data collection design of the MCS had numerous consequences for designing its questionnaires and choosing its measurement instruments. This included designing new instruments on the fly in the ever-changing pandemic environment, making efficient use of limited questionnaire space, and deciding on measurement frequencies in a structured manner under uncertain external conditions. In this report, we document the MCS approach to choosing measurement instruments fit for the purpose of fast and frequent data collection during the early phase of COVID-19 in Germany. We particularly highlight three examples of measurement instruments in the MCS and reflect on their measurement properties.

**Supplementary Information:**

The online version contains supplementary material available at 10.1186/s42409-022-00030-5.

## The pandemic and the Mannheim Corona Study (MCS)

Looking back at the early phase of COVID-19 in Germany (i.e., approximately the first half of 2020), we now know that people’s lives changed dramatically and repeatedly during that time and that the policies devised to combat the pandemic have had wide-ranging consequences (Naumann, Möhring, et al., 2020a). Among other aspects, the pandemic affected people’s employment situation (Möhring, Weiland, et al., 2021a), mental health (Mata et al., 2020), political attitudes (Juhl et al., 2020, forthcoming), and life satisfaction (Möhring, Naumann, et al., 2021b). Some of the evidence that we have on the societal impact of COVID-19 during the early phase of the pandemic in Germany has been contributed by the Mannheim Corona Study (MCS).

The MCS is based on the data collection infrastructure of the German Internet Panel (GIP). The GIP is a probability-based online panel of the general population in Germany. In 2020, it included participants of ages 18 to 83. To date, the GIP has seen three recruitment rounds: in 2012, 2014, and 2018 (Blom et al., 2015; Cornesse et al., 2021a, b). In the first two recruitment rounds, sample units without internet access were provided with the necessary equipment to participate in the GIP’s bi-monthly web surveys (Blom et al., 2017; Cornesse & Schaurer, 2021). The average length of the regular GIP survey waves is 20 to 25 min and respondents receive a 4€ conditional incentive per completed questionnaire (plus a 10€ bonus if they participate in all 6 survey waves of a year or a bonus of 5€ if they only miss one wave), which is credited to their panel accounts and paid out twice a year as online vouchers, bank transfers, or charitable donations depending on the panel member’s preferences.

During the early phase of the pandemic in Germany, the MCS was set up to study the societal impact of the COVID-19 pandemic from an interdisciplinary perspective. The diverse range of topics covered by the MCS included changes to people’s employment and financial situation, their childcare arrangements, satisfaction with the work of selected politicians, attitudes towards democratic processes, the frequency of people’s social interactions, and feelings of anxiety. For the MCS, the GIP participant sample was split into eight random sub-samples (Blom, Cornesse, et al., 2020a; Blom, Cornesse, et al., 2021a; Cornesse, Krieger, et al., 2021b). The first seven sub-samples were each assigned a day of the week. For the subsequent 16 weeks, the panel members in these sub-samples were invited via email to participate in a short survey on the weekday they were assigned to (e.g., sample members who were assigned to Monday received survey invitations each Monday). After each survey invitation, study participants had 48 h to complete the survey. However, they were encouraged to participate within the first 24 h of being invited. The eighth sub-sample served as a control group to study the impact of the data collection adaptation process on the GIP infrastructure. The average length of the surveys was 8 min and respondents received a 2€ conditional incentive per survey, which was credited towards their GIP panel accounts. On average, 3419 people participated in the MCS data collection each week. Since the MCS was piggy-backed on the GIP, previous GIP measurements, including detailed socio-demographics, were available as background information for the MCS sample.

## MCS measurement instruments

The frequent data collection as well as the short fieldwork times in the MCS required the survey questionnaires to be short to ensure panel participants’ continued and timely participation. However, gaining insights into multiple aspects of how the pandemic impacted society required a variety of different types of measurement instruments. Balancing the research aims against the practical questionnaire space restrictions resulted in 66 measurement instruments in the MCS questionnaires. Of these instruments, 14 were multiple-item batteries (e.g., measuring multi-dimensional latent constructs), while 52 of them were single-item instruments (e.g., measuring socio-demographic characteristics). Moreover, 32 instruments were either taken or slightly adapted from external sources (e.g., European Social Survey) and/or had previously already been fielded in the GIP, whereas 34 instruments were purposively developed for the MCS. Furthermore, 14 instruments measured behavior (e.g., adherence to COVID-19 protective measures) and 20 instruments measured objective facts (e.g., employment status), while 20 instruments measured attitudes (e.g., towards introducing legislature granting employees the right to work from home) and 12 instruments measured other subjective characteristics (e.g., fear of contracting the virus).

A few measurement instruments were expanded, changed, or reduced during the course of the MCS due to new societal or epidemiological developments or to make space for new instruments on topics that had recently gained relevance in the public debate. For example, a short-scale on state-trait-anxiety was reduced from its original five items to two items after four weeks to create space for new measurement instruments, whereas an item on support for tracking mobile phones was added to an item battery on support for various COVID-19 containment measures after the first week due to its increased relevance in the public debate.

Overall, the MCS measurement instruments consisted of 151 items of which 57 can be regarded as belonging to multiple-item measurement instruments (e.g., latent construct scales), and 94 are single-item instruments. Moreover, 70 items were only included during 1 week of data collection (i.e., the measurement was cross-sectional). Of these cross-sectional items, 13 were recall questions that asked about people’s situation before the pandemic (for a critical discussion of such retrospective questions, see Hipp et al., 2020). Apart from the cross-sectional items, 51 items were measured repeatedly (i.e., were included during more than 1 week of data collection to observe change, but less than 10 weeks in a row), and 30 items were continuously measured over time (i.e., they were either included every week or at least for 10 weeks in a row to allow tracking fine-grained changes over time).

All MCS questionnaires can be found here: https://www.uni-mannheim.de/en/gip/corona-study/questionnaires/. It should be noted that for some measurement instruments used in the MCS, we can draw comparisons to pre-pandemic times because the same instruments had been fielded in regular GIP survey waves (for an example of such an analysis see Möhring, Weiland, et al., 2021a). In the following, we provide three examples of different types of measurement instruments included in the MCS.

## Barriers to the adoption of contact tracing apps

**Fig. 1:**
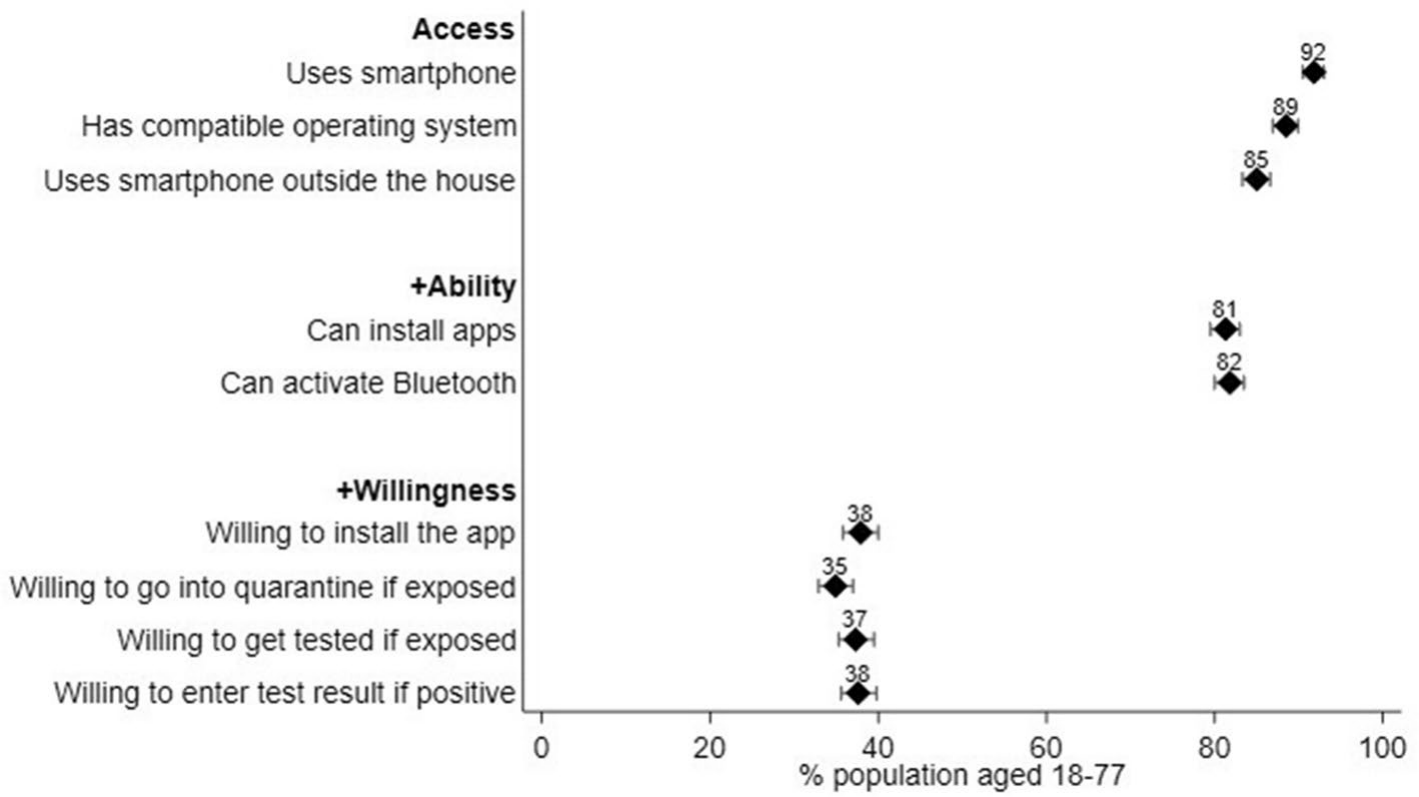
Predicted COVID-19 tracing app adoption rates by access, ability, and willingness. Error bars represent 95% confidence intervals; ©Annelies G Blom, Alexander Wenz, Carina Cornesse, Tobias Rettig, Marina Fikel, Sabine Friedel, Katja Möhring, Elias Naumann, Maximiliane Reifenscheid, Ulrich Krieger. Originally published in the Journal of Medical Internet Research (http://www.jmir.org), 02.03.2021

During the same week as the official German contact tracing app (“Corona-Warn-App”) was launched, we implemented a cross-sectional questionnaire module in the MCS to enable the prediction of potential barriers to the success of such an app. Among other aspects, the module included questions on people’s access, ability, and willingness to use the app (see Fig. 1, reproduced from Blom, Wenz, et al., 2021b; for question texts, see Table B1 in Additional file 2: Appendix B).

The questionnaire module was developed by a group of researchers within the MCS research group who brought together their existing expertise on people’s willingness to provide location data (Felderer & Blom, 2019), download and use smartphone apps (Wenz et al., 2019), and to use the internet in general (Cornesse & Schaurer, 2021). In discussions, these researchers developed the initial version of the questionnaire module. Their discussions were in part influenced by a previous study on the acceptance of app-based contact tracing conducted by Abeler and colleagues in March and April 2020 using nonprobability survey data. Some of the items used by Abeler et al. (2020), in particular items on smartphone use outside the home and willingness to install a contact tracing app, were adapted for the MCS questionnaire module. However, most items were newly developed for the particular research purpose of the MCS.

The initial version of the questionnaire was reviewed and discussed by the entire MCS research group, which included additional experts on a range of survey methodological and substantive social science topics. After revisions based on the expert comments (e.g., on question wording), the final version of the questionnaire module was fielded during the 13th week of the MCS (i.e., between June 12 and June 19, 2020). The overall result from the study was that the officially set objective of an app adoption rate of 56% in the German population would likely be missed by a great margin. What is more, among two relevant subgroups, potential spreaders and people with a high risk of infection, the adoption rate was expected to be no higher than in the general population (for detailed substantive analyses on this topic see Blom et al., 2021b).

Due to time constraints, the adapted and newly developed measures could not be pretested prior to being included in the MCS (e.g., using cognitive interviews and/or rapid online tests of draft survey items). However, we carefully introduced each question, providing context and definitions to help respondents answer the questions, avoided complex (e.g., technological) terminology and offered simple definitions where technological terminology could not be avoided (e.g., for the term “Bluetooth”).

We find evidence of moderately high to high scale reliability among the three access measures in the module (average inter-item correlation = 0.73, Cronbach’s alpha = 0.89), the two ability measures (inter-item correlation = 0.55, Cronbach’s alpha = 0.71), and the four willingness measures (average inter-item correlation = 0.51, Cronbach’s alpha = 0.80). Moreover, we find evidence of convergent validity when comparing people’s reported degree of willingness to install the app in the MCS module (measured on a fully labeled 5-point scale) to self-reports of these same people on whether they actually installed the app, which were gathered in the GIP three month after the MCS tracing app module (i.e., in September 2020). As can be expected, willingness to install the app in June 2020 correlates significantly and positively with actually installing the app by September 2020 (Spearman’s rank correlation coefficient = 0.54).

## State-Trait Anxiety Short Scale

**Fig. 2:**
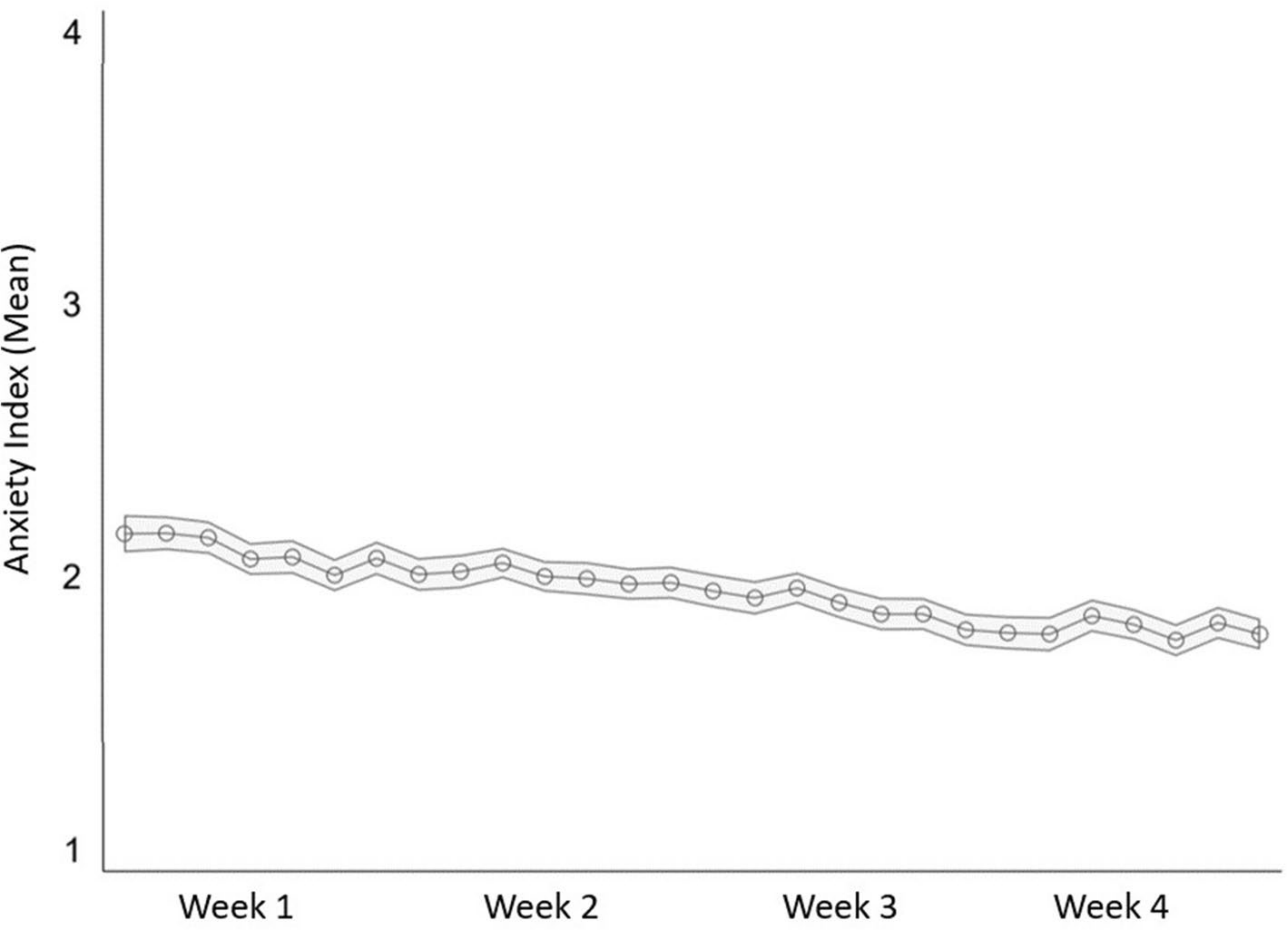
Mean anxiety levels across the first four MCS data collection week. The calculated anxiety scale ranges between 1 and 4. Light gray area represents 95% confidence intervals

During the first four weeks of the MCS, we repeatedly implemented a five-item short scale of the State-Trait Anxiety Inventory (STAI-SKD) developed for the German context by Englert et al., 2011(see Fig. 2 adapted from Naumann, Mata, et al., 2020b).

The full STAI-SKD scale was fielded from the first until the fourth week of the MCS (i.e., between March 20 and April 17, 2020). Findings generally indicate a small but steady decrease in anxiety over time (the scale of the pseudo-metric additive index depicted in Fig. 2 ranges from 1 to 4). After week 4, three of the STAI-SKD items were discontinued (feeling tense, agitated, and disturbed), while two items (feeling worried and nervous) remained in the MCS questionnaires until the end of the study in July 2020. This decision was taken to make space for new measurement instruments after week 4.

Englert et al. (2011) specifically adapted the STAI-SKD from the established longer STAI scale (Laux et al., 1981; Spielberger et al., 1970) for purposes such as the MCS (i.e., taking repeated measures of anxiety in situations where questionnaire space is limited). In addition, the authors thoroughly constructed and validated the scale in three separate studies, using among other techniques, a confirmatory factor analysis (CFA).

Generally, we find moderate to high test-retest reliability of the STAI-SKD across data collection weeks. Intraclass correlation coefficients (ICC) across the four measurement time points are 0.59 (item: feeling tense), 0.66 (agitated), 0.61 (worried as well as disturbed), and 0.68 (nervous; see Table A2 for more information). This is in line with the theory on the STAI, which postulates that the inventory has a time-stable as well as a situationally changeable component (Englert et al., 2011). Furthermore, when we replicate the CFA conducted by Englert and colleagues, we find evidence of high construct validity. The Comparative Fit Index (CFI), Tucker-Lewis Index (TLI), and Standardized Root Mean Square Residual (SRMR) indicate good model fit (CFI ≥ 0.98, TLI ≥ 0.95, SRMR = 0.02 at all four data collection weeks; see Table A1). It should be noted, however, that chi-squared statistics and root mean square error of approximations (RMSEA) suggest poor model fit (*p*-values of chi-squared statistics are < 0.01 and RMSEA values are between 0.11 and 0.12 at all data collection weeks). This is presumably an artifact, as the chi-squared statistic is sensitive to large sample sizes (in our study: between 3362 and 3602 cases per measurement time point; Hooper et al., 2008) while the RMSEA is sensitive to low degrees of freedom (in our study: df = 4; Kenny et al., 2015).

## Attitudes towards COVID-19 political measures

**Fig. 3:**
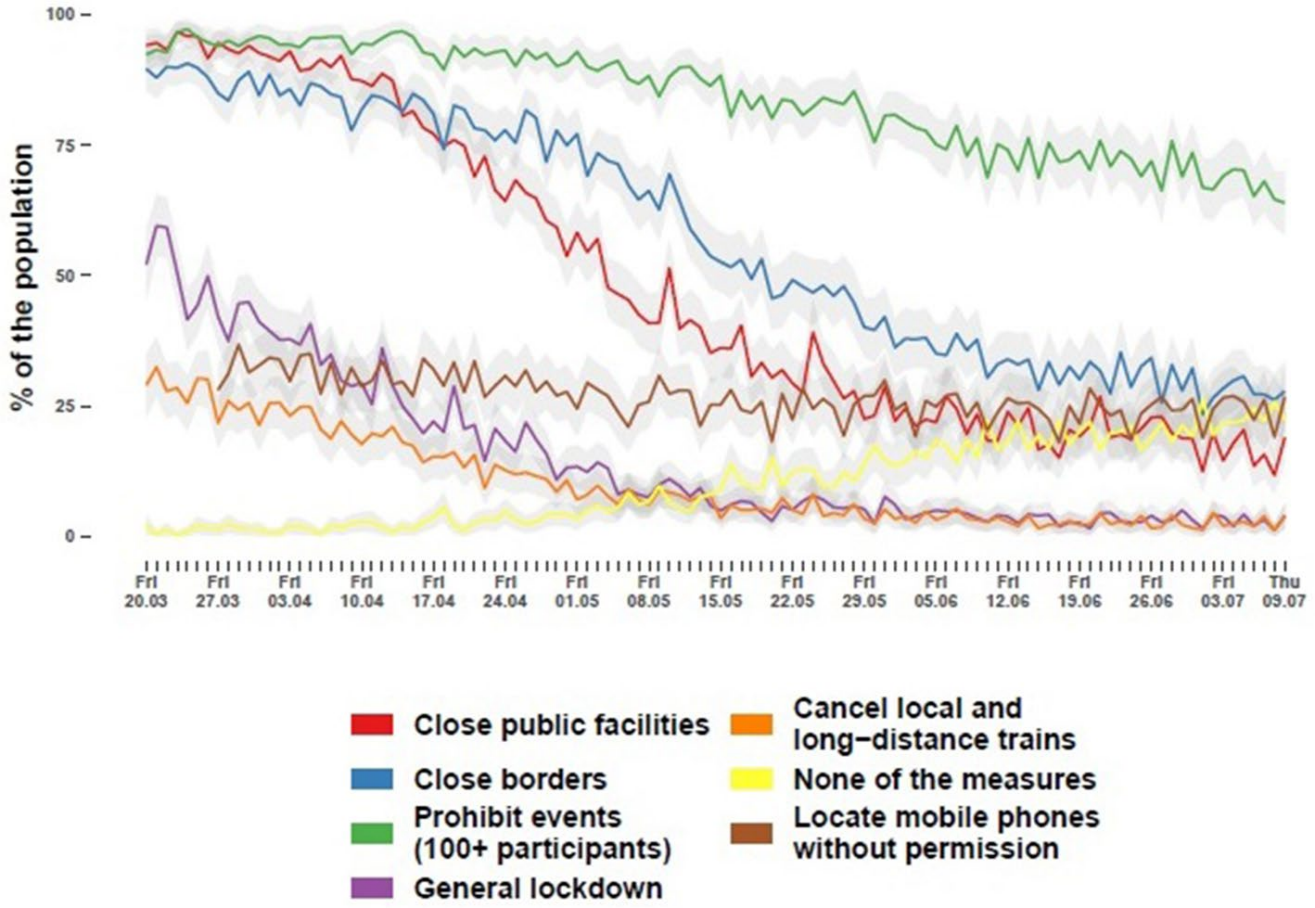
Proportion of the population that endorses certain measures on the day of the survey. Light gray areas represent 95% confidence intervals

While cross-sectional and repeated-measures instruments make up an important part of the MCS questionnaires, measuring fine-grained changes to people’s lives using longitudinal panel data instruments is a particularly valuable part of the MCS. One example of this is a battery on support for a range of COVID-19 political measures to contain the spread of the pandemic (see Fig. 3 reproduced from Blom, Wenz, et al., 2020b). The battery was developed right at the start of the MCS by experts on repeated survey measurement (Rettig & Blom, 2021), attitude formation and change (Moehring & Teney, 2021; Naumann, 2017), and citizens’ perceptions of politics (Juhl et al., forthcoming; Lehrer et al., 2021). To include all relevant items in the battery, the researchers conducted a thorough review of the public discussions on containment measures at the time the MCS was set up (i.e., March 2020). The researchers chose to include the five COVID-19 containment measures for the battery that were at that time already being implemented to at least some extent either nationally or locally (i.e., closing universities, schools and childcare facilities, closing borders, banning events with more than 100 people, general lockdown, stop local and long-distance transport). They also chose to offer participants the option to state that they did not support any of the COVID-19 containment measures.

The battery was implemented and introduced to the respondents as a multiple-choice question. The implementation included a plausibility check that did not allow respondents who chose to support at least one containment measure to additionally check the box stating that they supported none of the measures and vice versa. After the first week of data collection, an item on tracking mobile phones was added to the battery, because it had gained relevance in the public debate during that week. In hindsight, it may have been valuable to continue adding further measures (e.g., support for mask mandates) throughout the course of the MCS. At the time, however, it seemed unclear which of the multitude of potential containment measures would become and remain relevant.

A wide array of findings was retrieved from this item battery. For example, support for most measures declined over the course of the MCS, in particular for those measures that were strongly supported in the beginning (for more detailed findings see e.g., Juhl et al., 2020, forthcoming).

As can be seen in Fig. 3, attitudes towards the containment measures changed a lot over time, resulting in low test-retest reliability across data collection weeks (ICC > 0.25 and < 0.36 on all items measured 16 weeks in a row; see Table A2). An exception is the item on support for tracking phones, which was added to the battery later, and where test-retest reliability is relatively high (ICC = 0.70). However, we find evidence of test-retest reliability when examining inter-item correlations across data collection weeks. Generally, average inter-item correlations across the substantive items of the battery range only between 0.15 and 0.18 across data collection weeks and Cronbach’s alpha ranges only between 0.52 and 0.57. This suggests that, while attitudes change over time, the relation among the items of the battery is highly stable. Furthermore, we find moderate evidence of construct validity when examining the correlation between supporting at least one of the containment measures with the degree to which respondents perceive COVID-19 as a threat. The latter instrument was measured in the MCS from the fifth week of data collection until the end of the study. This correlation between the containment measure battery and the COVID-19 perceived threat instrument is significantly positive at all the available measurement time points, albeit at a moderate level (Pearson’s point-biserial correlation coefficients range between 0.10 and 0.24 across data collection weeks).

## Discussion and conclusion

The early phase of the COVID-19 pandemic was characterized by high volatility and high uncertainty in people’s lives. Capturing the diverse societal developments during that time required fast-and-frequent data collection designs, such as the one used in the MCS. In this report, we give an overview and provide examples of the measurement instruments used in the MCS to document the special circumstances encountered when aiming to help meet the urgent data demand during the early phase of the pandemic.

In sum, we believe that the mix of cross-sectional, repeated-measures, and longitudinal panel measurements provided a good balance for studying a wide variety of societal impacts of the pandemic. In addition, we believe that the mix of replicated or adapted and newly developed measurement instruments provided a good balance between confidence in tried and tested instruments and paying credit to the need to ask questions that, before the beginning of the pandemic, had never been on researcher’s minds.

One limitation of the approach described in this paper is that the need to develop and directly field new measurement instruments left no time to test these new measurement instruments beforehand, for example using cognitive interviews or rapid online tests of draft questions (ideally including web probing questions, see e.g., Meitinger & Behr, 2016). Such pretest approaches would have helped to ensure that respondents comprehend the questions correctly, feel able to retrieve the relevant information from their memories, make reasonable judgments to arrive at an answer, and feel that the offered answer options allow them to report their answers adequately (Tourangeau et al., 2000). Moreover, while we tried to react to new developments in the course of the MCS by adapting the questionnaires, some aspects did not receive as much attention as may have been desirable in hindsight, given the knowledge about the pandemic that we have today. This has arguably let to incomplete measurement instruments over the course of the MCS, which is evident in the containment measure battery missing an item on the now prevalent mask mandates, which were not a vital part of the public discussions in March 2020, but became important during the course of the MCS study period.

## Supplementary Information

**Additional file 1: Appendix A: Table A1.** Results of confirmatory factor analysis on STAI-SKD. **Table A2.** Test-retest reliability of the STAI-SKD.

**Additional file 2: Appendix B: Table B1.** Question and answer texts of selected items on contact tracing apps. **Table B2.** Question and answer texts of STAI-SKD scale (unofficial translation). **Table B3.** Question and answer texts of COVID-19 political measures support scale.

## Data Availability

MCS data are available from the GESIS Data Archive (10.4232/1.13700).

## References

[CR1] Abeler, J., Altmann, S., Bach, R., Gerdon, F., Kreuter, F., & Milsom, L. (2020). Akzeptanz App-basierter Kontaktnachverfolgung von Covid-19. Retrieved from https://osf.io/z6ws4/.

[CR2] Blom, A. G., Cornesse, C., Friedel, S., Krieger, U., Fikel, M., Rettig, T., … Reifenscheid, M. (2020a). High-frequency and high-quality survey data collection. *Survey Research Methods*, *14*(2), 171–178 10.18148/srm/2020.v14i2.7735.

[CR3] Blom, A. G., Cornesse, C., Friedel, S., Krieger, U., Fikel, M., Rettig, T., Wenz, A., Juhl, S., Lehrer, R., Möhring, K., Naumann, E. & Reifenscheid, M. (2021a). Mannheim Corona Study. GESIS Data Archive, Cologne. ZA7745 Data file Version 1.0.0. 10.4232/1.13700.

[CR4] Blom AG, Gathmann C, Krieger U (2015). Setting up an online panel representative of the general population: the German Internet Panel. Field Methods.

[CR5] Blom AG, Herzing JM, Cornesse C, Sakshaug JW, Krieger U, Bossert D (2017). Does the recruitment of offline households increase the sample representativeness of probability-based online panels? Evidence from the German Internet Panel. Social Science Computer Review.

[CR6] Blom, A. G., Wenz, A., Cornesse, C., Rettig, T., Fikel, M., Friedel, S., … Krieger, U. (2021b). Barriers to the large-scale adoption of the COVID-19 contact-tracing app in Germany: Survey study. *Journal of Medical Internet Research*, 23(3), e23362. 10.2196/23362. 10.2196/23362 PMC7927947 33577466

[CR7] Blom, A. G., Wenz, A., Rettig, T., Reifenscheid, M., Naumann, E., Möhring, K., Lehrer, R., Krieger, U., Juhl, S., Friedel, S., Fikel, M., & Cornesse, C. (2020b). The Mannheim Corona study: life in Germany in a state of emergency: Report for March 20 to July 09, 2020. Retrieved from https://madoc.bib.uni-mannheim.de/55629.

[CR8] Cornesse, C., Felderer, B., Fikel, M., Krieger, U., & Blom, A. G. (2021a). Recruiting a probability-based online panel via postal mail: experimental evidence. *Social Science Computer Review. Advance access via*. 10.1177/08944393211006059.

[CR9] Cornesse, C., Krieger, U., Sohnius, M. L., Fikel, M., Friedel, S., Rettig, T., Wenz, A., Juhl, S., Lehrer, R., Möhring, K., Naumann, E., Reifenscheid, M. & Blom, A. G. (2021b). From German Internet Panel to Mannheim Corona Study: adaptable probability-based online panel infrastructures during the pandemic. *Journal of the Royal Statistical Society: Series A (Statistics in Society)*. Advance access via 10.1111/rssa.12749.

[CR10] Cornesse, C., & Schaurer, I. (2021). The long-term impact of different offline population inclusion strategies in probability-based online panels: evidence from the German Internet Panel and the GESIS Panel. *Social Science Computer Review. Advance access via*. 10.1177/0894439320984131.

[CR11] Englert C, Bertrams A, Dickhäuser O (2011). Entwicklung der Fünf-Item-Kurzskala STAI-SKD zur Messung von Zustandsangst. Zeitschrift für Gesundheitspsychologie.

[CR12] Felderer, B., & Blom, A. G. (2019). Acceptance of the automated online collection of geographical information. *Sociological Methods & Research. Advance access via*. 10.1177/0049124119882480.

[CR13] Hipp L, Bünning M, Munnes S, Sauermann A (2020). Problems and pitfalls of retrospective survey questions in COVID-19 studies. Survey Research Methods.

[CR14] Hooper D, Coughlan J, Mullen MR (2008). Structural equation modelling: guidelines for determining model fit. Electronic Journal of Business Research Methods.

[CR15] Juhl, S., Lehrer, R., Blom, A. G., Wenz, A., Rettig, T., Krieger, U., ... & Möhring, K. (2020). Determinants of public support for COVID-19 containment policies in Germany: evidence from individual-level panel analyses. Retrieved from http://www.ronilehrer.com/docs/Who_support_COVID_measures.pdf.

[CR16] Juhl, S., Lehrer, R., Blom, A. G., Wenz, A., Rettig, T., Krieger, U., Fikel, M., Cornesse, C., Naumann, E., Möhring, K. & Reifenscheid, M. (forthcoming). Preferences for centralized decision-making in times of crisis: the COVID-19 pandemic in Germany. In: Debus, M., Tepe, M. &Sauermann, J. (eds.). *Jahrbuch für Handlungs- und Entscheidungstheorie.* Retrieved from http://ronilehrer.com/docs/JHET.pdf.

[CR17] Kenny DA, Kaniskan B, McCoach DB (2015). The performance of RMSEA in models with small degrees of freedom. Sociological Methods & Research.

[CR18] Laux L, Glanzmann P, Schaffner P, Spielberger CD (1981). *Das State-Trait-Angstinventar*.

[CR19] Lehrer, R., Bahnsen, O., Müller, K., Neunhöffer, M., Gschwend, T., & Juhl, S. (2021). Crisis leadership approval: the opposing effects of perceived threat and anxiety. Retrieved from https://www.sowi.uni-mannheim.de/media/Lehrstuehle/sowi/Gschwend/Articel/Crisis_Leadership_Approval_The_Opposing_Effects_of_Perceived_Threat_and_Anxiety.pdf.

[CR20] Mata, J., Wenz, A., Rettig, T., Reifenscheid, M., Moehring, K., Krieger, U., … Naumann, E. (2020). Health behaviors and mental health before and during the COVID-19 pandemic: a longitudinal population-based survey. *Social Science and Medicine 287*, 114333. 10.1016/j.socscimed.2021.114333. 10.1016/j.socscimed.2021.114333 PMC8479385 34455337

[CR21] Meitinger K, Behr D (2016). Comparing cognitive interviewing and online probing: do they find similar results?. Field Methods.

[CR22] Moehring K, Teney C (2021). Who supports affirmative action policies for women and immigrants in recruitment processes?. An international survey experiment.

[CR23] Möhring, K., Naumann, E., Reifenscheid, M., Wenz, A., Rettig, T., Krieger, U., ... & Blom, A. G. (2021b). The COVID-19 pandemic and subjective well-being: longitudinal evidence on satisfaction with work and family. *European Societies*, 23(sup1), S601-S617. 10.1080/14616696.2020.1833066

[CR24] Möhring, K., Weiland, A., Reifenscheid, M., Naumann, E., Wenz, A., Rettig, T., … Blom, A. G. (2021a). Inequality in employment trajectories and their socio-economic consequences during the early phase of the COVID-19 pandemic in Germany. *Retrieved from*. 10.31235/osf.io/m95df.

[CR25] Naumann, E. (2017). Do increasing reform pressures change welfare state attitudes? An experimental study on population ageing, pension reform preferences, political knowledge and ideology. *Ageing & Society*, *37*(2), 266–294.

[CR26] Naumann, E., Mata, J., Reifenscheid, M., Möhring, K., Wenz, A., Rettig, T., ... & Blom, A. G. (2020b). Die Mannheimer Corona-Studie: Schwerpunktbericht zum Angstempfinden in der Bevölkerung. Retrieved from https://madoc.bib.uni-mannheim.de/55136/.

[CR27] Naumann, E., Möhring, K., Reifenscheid, M., Wenz, A., Rettig, T., Lehrer, R., … Blom, A. G. (2020a). COVID-19 policies in Germany and their social, political, and psychological consequences. *European Policy Analysis, 6*(2), 191–202. 10.1002/epa2.1091. 10.1002/epa2.1091 PMC7537296 34616900

[CR28] Rettig T, Blom AG, Cernat A, Sakshaug JW (2021). Memory Effects as a Source of Bias in Repeated Survey Measurement. *Measurement Error in Longitudinal Data*.

[CR29] Spielberger CD, Gorsuch RL, Lushene RE (1970). *Manual for the State-Trait Anxiety Inventory*.

[CR30] Tourangeau R, Rips LJ, Rasinski K (2000). *The psychology of survey response*.

[CR31] Wenz A, Jäckle A, Couper MP (2019). Willingness to use mobile technologies for data collection in a probability household panel. Survey Research Methods.

