# Supplementary material for: Measurement instruments for fast and frequent data collection during the early phase of COVID-19 in Germany: reflections on the Mannheim Corona Study
Source: Meas Instrum Soc Sci. 2022 Feb 22;4(1):2. doi: 10.1186/s42409-022-00030-5 (PMC8861594; doi:10.1186/s42409-022-00030-5)
Supplement: Supplementary file 1 — Additional file 1: Appendix A: Table A1. Results of confirmatory factor analysis on STAI-SKD. Table A2. Test-retest reliability of the STAI-SKD. [file 42409_2022_30_MOESM1_ESM.docx]

# Appendix A: Additional tables

**Table A1** Results of confirmatory factor analysis on STAI-SKD

|  | **Week 1**  (N=3,362) | | **Week 2**  (N=3,602) | | **Week 3**  (N=3,559) | | **Week 4**  (N=3,516) | |
| --- | --- | --- | --- | --- | --- | --- | --- | --- |
|  | **Unst.**  **(SE)** | **Std.**  **(SE)** | **Unstd.**  **(SE)** | **Std.**  **(SE)** | **Unst.**  **(SE)** | **Std.**  **(SE)** | **Unstd.**  **(SE)** | **Std.**  **(SE)** |
| Emotionality – tense | 1.00  (—) | 0.79  (0.01) | 1.00  (—) | 0.82  (0.01) | 1.00  (—) | 0.81  (0.01) | 1.00  (—) | 0.83  (0.01) |
| Emotionality – agitated | 1.03  (0.20) | 0.82  (0.03) | 1.03  (0.02) | 0.85  (0.01) | 1.05  (0.02) | 0.88  (0.01) | 1.03  (0.02) | 0.89  (0.00) |
| Emotionality – nervous | 1.14  (0.02) | 0.85  (0.01) | 1.14  (0.02) | 0.88  (0.01) | 1.08  (0.02) | 0.88  (0.01) | 1.06  (0.02) | 0.89  (0.01) |
| Worry – worried | 1.00  (—) | 0.81  (0.01) | 1.00  (—) | 0.82  (0.01) | 1.00  (—) | 0.81  (0.01) | 1.00  (—) | 0.80  (0.01) |
| Worry – disturbed | 1.09  (0.02) | 0.86  (0.01) | 1.09  (0.02) | 0.88  (0.01) | 1.16  (0.02) | 0.90  (0.01) | 1.18  (0.02) | 0.90  (0.01) |
| **Correlations among latent variables** | | | | | | | | |
| Emotionality – Worry | 0.35  (0.01) | 0.89  (0.01) | 0.36  (0.01) | 0.89  (0.01) | 0.32  (0.01) | 0.87  (0.01) | 0.31  (0.01) | 0.88  (0.01) |
| **Global fit measures** | | | | | | | | |
| χ^2^(4) | 157.81  *p=*0.000 | | 224.74  *p=*0.000 | | 187.56  *p=*0.000 | | 193.70  *p=*0.000 | |
| RMSEA | 0.11  *p=*0.000 | | 0.12  *p=*0.000 | | 0.11  *p=*0.000 | | 0.12  *p=*0.000 | |
| CFI | 0.98 | | 0.98 | | 0.99 | | 0.99 | |
| TLI | 0.96 | | 0.95 | | 0.96 | | 0.99 | |
| SRMR | 0.02 | | 0.02 | | 0.02 | | 0.02 | |

*Note. We report unstandardized (unst.) and standardized (std) factor loadings as well as their respective standard errors (SE).*

**Table A2.** Test-retest reliability of the STAI-SKD

| **Measure** | **Intraclass correlation** | **95% Confidence interval** |
| --- | --- | --- |
| STAI-SKD |  |  |
| Tense  (n = 2,751; t = 4) | 0.59 | 0.55 - 0.63 |
| Agitated  (n = 2,753; t = 4) | 0.66 | 0.64 - 0.68 |
| Worried  (n = 1,901; t = 16) | 0.64 | 0.61 - 0.66 |
| Disturbed  (n = 2,754; t = 4) | 0.61 | 0.56 - 0.65 |
| Nervous  (n = 1,902; t = 16) | 0.68 | 0.67 - 0.70 |
| COVID-19 containment measures |  |  |
| Closing public facilities  (n = 1,895; t = 16) | 0.25 | 0.18 - 0.32 |
| Closing borders  (n = 1,895; t = 16) | 0.33 | 0.27 - 0.40 |
| Ban events >100 people  (n = 1,895; t = 16) | 0.37 | 0.24 - 0.32 |
| General lockdown  (n = 1,895; t = 16) | 0.28 | 0.24 - 0.32 |
| Stop public transport  (n = 1,895; t = 16) | 0.32 | 0.29 - 0.34 |
| Track phones  (n = 1,994; t = 15) | 0.70 | 0.69 - 0.72 |
| None of the measures  (n = 1,895; t = 16) | 0.36 | 0.89 - 0.91 |

*Note. The intra-class correlation coefficients are based on two-way mixed-effects models and* *provide evidence of the absolute agreement in respondents’ answers to the questionnaire items across measurement time points (t).*
