# Supplementary material for: Measurement instruments for fast and frequent data collection during the early phase of COVID-19 in Germany: reflections on the Mannheim Corona Study
Source: Meas Instrum Soc Sci. 2022 Feb 22;4(1):2. doi: 10.1186/s42409-022-00030-5 (PMC8861594; doi:10.1186/s42409-022-00030-5)
Supplement: Supplementary file 2 — Additional file 2: Appendix B: Table B1. Question and answer texts of selected items on contact tracing apps. Table B2. Question and answer texts of STAI-SKD scale (unofficial translation). Table B3. Question and answer texts of COVID-19 political measures support scale. [file 42409_2022_30_MOESM2_ESM.pdf]

Appendix B. Selected question and answer texts

Table B1 Question and answer texts of selected items on contact tracing apps

German	English
Im Folgenden stellen wir Ihnen ein paar Fragen zur Nutzung der offiziellen Corona-Warn-App, die bald verfügbar sein soll. Vielleicht haben Sie in den Medien bereits davon gehört.<br><br>Die offizielle Corona-Warn-App wird vom Robert-Koch-Institut (RKI) zur Verfügung gestellt. Das RKI ist die zentrale Einrichtung der Bundesregierung auf dem Gebiet der Krankheitsüberwachung und -prävention. Die Corona-Warn-App kann dazu beitragen, die Ausbreitung der Corona Pandemie einzudämmen, indem sie Kontaktpersonen von Infizierten über eine mögliche Infektion informiert.<br><br>Wir möchten herausfinden, inwieweit die Einführung dieser App effektiv bei der Eindämmung der Corona-Pandemie sein wird. Deshalb wollen wir Ihnen gerne Fragen darüber stellen, ob Sie die nötigen Geräte und Kenntnisse haben, um die App zu installieren und zu nutzen, und ob Sie die App auf Ihrem Smartphone installieren und nutzen möchten.<br><br>Es gibt keine richtigen und falschen Antworten. Wir sind an Ihren Erfahrungen und Ihren Meinungen interessiert.	In the following, we ask you several questions about the use of the official Corona-Warn-App that should be available soon. Maybe you have already heard about it through the media.<br><br>The official Corona-Warn-App is made available by the Robert-Koch-Institute (RKI). The RKI is the central institution of the federal government in the area of disease control and prevention. The Corona-Warn-App can help contain the spread of the corona pandemic by informing persons, who have been in contact with someone infected, of a potential infection.<br><br>We aim to find out to what extent the introduction of this app will be effective at containing the corona pandemic. Therefore, we would like to ask you questions about whether you have the necessary devices and knowledge to install and use the app, and whether you would be willing to install and use the app on your smartphone.<br><br>There are no right and wrong answers. We are interested in your experiences and your opinions
Um die offizielle Corona-Warn-App nutzen zu können, benötigen Sie ein Smartphone, d.h. ein Mobiltelefon, das Internetzugang hat und auf dem Apps, also zusätzliche Programme, installiert werden können.<br><br>**Nutzen Sie persönlich ein Smartphone?**<br><br>1 Ja, ich nutze ein Smartphone.<br>2 Nein, ich nutze kein Smartphone.<br>3 Ich nutze ein Mobiltelefon, bin mir aber nicht sicher, ob das ein Smartphone ist.	To be able to use the official Corona-Warn-App you need a smartphone, i.e. a cell phone that has Internet access and on which apps, i.e. additional programs, can be installed.<br><br>**Do you personally use a smartphone?**<br><br>1 Yes, I use a smartphone.<br>2 No, I don't use a smartphone.<br>3 I use a cell phone but am not sure whether it is a smartphone.
(wenn Befragte/-r Smartphone nutzt)<br><br>**Welcher der folgenden Typen beschreibt Ihr Smartphone am besten?**	(If respondent uses a smartphone)<br><br>**Which of the following types best describes your smartphone?**

Wenn Sie mehrere Smartphones nutzen, dann beantworten Sie diese Frage bitte für das Smartphone, das Sie am häufigsten nutzen.<br><br>1 iPhone<br>2 Android Phone<br>3 Etwas anderes, und zwar: [answer field] 4 Ich weiß es nicht.	If you use several smartphones, then please answer this question for the smartphone you use most often.<br><br>1 iPhone<br>2 Android Phone<br>3 Something else: [answer field]<br>4 I am not sure
(wenn Befragte/-r Smartphone nutzt)<br><br>**Wie häufig haben Sie Ihr Smartphone dabei, wenn Sie das Haus verlassen?**<br><br>1 Immer<br>2 Meistens<br>3 Manchmal<br>4 Selten<br>5 Nie	(If respondent uses a smartphone)<br><br>**How often do you carry your smartphone with you when you leave the house?**<br><br>1 Always<br>2 Most of the time<br>3 Sometimes<br>4 Rarely<br>5 Never
(wenn Befragte/-r Smartphone nutzt)<br><br>Die offizielle Corona-Warn-App können Sie im Apple App Store oder im Google Play Store herunterladen und auf Ihrem Smartphone installieren.<br><br>**Wissen Sie, wie man eine App, also ein zusätzliches Programm, auf Ihrem Smartphone installiert?**<br><br>1 Ja<br>2 Nein<br>3 Ich bin mir nicht sicher.	(If respondent uses a smartphone)<br><br>You can download the official Corona-Warn-App in the Apple App Store or in the Google Play Store and install it on your smartphone.<br><br>**Do you know how to install an app, i.e., an additional program, on your smartphone?**<br><br>1 Yes<br>2 No<br>3 I am not sure.
(wenn Befragte/-r nicht weiß wie man eine App installiert oder sich nicht sicher ist)<br><br>**Kennen Sie jemanden, der Ihnen beim Installieren der Corona-Warn-App auf Ihrem Smartphone helfen könnte, z.B. Familie, Freunde oder Nachbarn?**<br><br>1 Ja<br>2 Nein<br>3 Ich bin mir nicht sicher.	(If respondent does not know how to install an app or is not sure)<br><br>**Do you know anyone who could help you with installing the Corona-Warn-App on your smartphone, e.g., family, friends or neighbors?**<br><br>1 Yes<br>2 No<br>3 I am not sure.
(wenn Befragte/-r Smartphone nutzt)<br><br>Sobald die Corona-Warn-App installiert wurde, wird die App feststellen, welche anderen Nutzer der App in Ihrer Nähe sind. Hierfür wird die App nicht auf Ihren Standort zugreifen, sondern Bluetooth nutzen, eine Technologie, die Daten per Funk zwischen zwei Geräten überträgt.	(If respondent uses a smartphone)<br><br>Once the Corona-Warn-App has been installed, the app will determine which other users of the app are in your proximity. To do this, the app will not access your position, but will use Bluetooth, a technology that transfers data via radio signal between two devices.

**Wissen Sie, wie man Bluetooth auf Ihrem Smartphone aktiviert?**<br><br>1 Ja<br>2 Nein<br>3 Ich bin mir nicht sicher.	**Do you know how to activate Bluetooth on your smartphone?**<br><br>1 Yes<br>2 No<br>3 I am not sure.
(wenn Befragte/-r nicht weiß wie man Bluetooth aktiviert oder sich nicht sicher ist)<br><br>**Kennen Sie jemanden, der Ihnen beim Aktivieren von Bluetooth auf Ihrem Smartphone helfen könnte, z.B. Familie, Freunde oder Nachbarn?**<br><br>1 Ja<br>2 Nein<br>3 Ich bin mir nicht sicher.	(If respondent does not know how to activate Bluetooth or is not sure)<br><br>**Do you know anyone who could help you with activating Bluetooth on your smartphone, e.g., family, friends or neighbors?**<br><br>1 Yes<br>2 No<br>3 I am not sure.
(wenn Befragte/-r nicht weiß wie man Bluetooth aktiviert oder sich nicht sicher ist)<br><br>Die Corona-Warn-App wird Sie automatisch benachrichtigen, falls Sie Kontakt zu jemandem gehabt haben, der mit dem Coronavirus infiziert ist. Dabei werden Sie nicht erfahren, wer die infizierte Person ist.<br><br>Dafür tauscht Ihr Smartphone verschlüsselte Identifikationsnummern mit anderen Smartphones aus, wenn die Smartphones längere Zeit nahe beieinander waren.<br><br>Die Nutzung der App ist kostenlos, freiwillig und entspricht den geltenden Datenschutzgesetzen und -bestimmungen. Sie soll in Deutschland schon diesen Monat zur Verfügung gestellt werden.<br><br>**Würden Sie die offizielle Corona-Warn-App auf Ihrem Smartphone installieren, wenn sie zur Verfügung steht?**<br><br>1 Auf jeden Fall installieren<br>2 Wahrscheinlich installieren<br>3 Vielleicht installieren, vielleicht nicht installieren<br>4 Wahrscheinlich nicht installieren<br>5 Auf keinen Fall installieren	(If respondent does not know how to activate Bluetooth or is not sure)<br><br>The Corona-Warn-App will automatically notify you, if you have been in contact with someone who is infected with the coronavirus. In this process, you will not be informed about who the infected person is.<br><br>For this purport, your smartphone exchanges encrypted identification numbers with other smartphones if the smartphones have been in close proximity to each other for a longer period.<br><br>Using the app is free, voluntary and meets the pertinent data protection laws and regulations. The app is meant to be made available in Germany already this month.<br><br>**Would you install the official Corona-Warn-App on your smartphone when it is available?**<br><br>1 Definitely install<br>2 Probably install<br>3 Maybe install, maybe not install<br>4 Probably not install<br>5 Definitely not install
(wenn Befragte/-r Smartphone besitzt und wenn Befragte/-r nicht „Auf keinen Fall installieren“ angegeben hat)	(If respondent uses a smartphone and if respondent has not answered “Definitely not install”)

Falls Sie sich an einer Kontaktperson infiziert haben könnten, werden Sie über die Corona-Warn-App gebeten, sich vorsorglich in häusliche Quarantäne zu begeben und sich auf das Virus testen zu lassen.<br><br>**Würden Sie der Bitte der Corona-Warn-App nachkommen, sich vorsorglich in häusliche Quarantäne zu begeben?**<br><br>*Personen in häuslicher Corona-Quarantäne müssen für ein bis zwei Wochen zu Hause bleiben und den Kontakt zu anderen Personen komplett einstellen.*<br><br>1 Auf jeden Fall nachkommen<br>2 Wahrscheinlich nachkommen<br>3 Vielleicht nachkommen, vielleicht nicht nachkommen<br>4 Wahrscheinlich nicht nachkommen<br>5 Auf keinen Fall nachkommen	If you might have been infected by a contact person, you will be asked through the Corona-Warn-App to go into precautionary domestic quarantine and to get tested for the virus.<br><br>**Would you comply with the request of the Corona-Warn-App to go into precautionary domestic quarantine?**<br><br>*Persons in domestic quarantine must stay at home for one to two weeks and cease contact with other persons completely.*<br><br>1 Definitely comply<br>2 Probably comply<br>3 Maybe comply, maybe not comply<br>4 Probably not comply<br>5 Definitely not comply
(wenn Befragte/-r Smartphone besitzt und wenn Befragte/-r nicht „Auf keinen Fall installieren“ angegeben hat)<br><br>**Würden Sie der Bitte der Corona-Warn-App nachkommen, sich auf das Virus testen zu lassen?**<br><br>*Um Sie auf das Coronavirus zu testen, entnimmt ein Arzt/eine Ärztin eine Probe aus den oberen oder tiefen Atemwegen, zum Beispiel als Abstrich aus dem Nasen-, Mund-, Rachenbereich oder dem abgehusteten Sekret und schickt die Probe anschließend an ein Labor.*<br><br>1 Auf jeden Fall nachkommen<br>2 Wahrscheinlich nachkommen<br>3 Vielleicht nachkommen, vielleicht nicht nachkommen<br>4 Wahrscheinlich nicht nachkommen<br>5 Auf keinen Fall nachkommen	(if respondent uses a smartphone and if respondent has not answered “Definitely not install”)<br><br>**Would you comply with the request of the Corona-Warn-App to get tested for the virus?**<br><br>*To get tested for the coronavirus, a doctor takes a sample from the upper or deeper respiratory tracts, for example a swab from your nose, mouth or throat or from coughed up secretion, and subsequently sends the sample to a laboratory.*<br><br>1 Definitely comply<br>2 Probably comply<br>3 Maybe comply, maybe not comply<br>4 Probably not comply<br>5 Definitely not comply
(wenn Befragte/-r Smartphone besitzt und wenn Befragte/-r nicht „Auf keinen Fall installieren“ angegeben hat)<br><br>Wenn Sie positiv auf das Virus getestet wurden, können Sie dies in der Corona-Warn-App eintragen.<br><br>Dadurch werden Ihre Kontaktpersonen benachrichtigt, dass sie sich angesteckt haben	(if respondent uses a smartphone and if respondent has not answered “Definitely not install”)<br><br>If you were tested positive for the virus, you can enter the test result into the Corona-Warn-App.<br><br>By doing this, your contact persons will be notified that they might have got infected. Your contact persons are asked to go into

könnten. Ihre Kontaktpersonen werden gebeten, sich vorsorglich in häusliche Quarantäne zu begeben und sich auf das Virus testen zu lassen.<br><br>Dass es sich bei der infizierten Person um Sie handelt, wird nicht weitergegeben; Sie bleiben also anonym.<br><br>**Würden Sie in der Corona-Warn-App eintragen, wenn Sie positiv auf das Virus getestet wurden?**<br><br>1 Auf jeden Fall eintragen<br>2 Wahrscheinlich eintragen<br>3 Vielleicht eintragen, vielleicht nicht eintragen<br>( ) Wahrscheinlich nicht eintragen<br>4 Auf keinen Fall eintragen	precautionary domestic quarantine and get tested for the virus.<br><br>The information that you are the infected person will not be passed on; you thus remain anonymous.<br><br>**Would you enter the test result into the Corona-Warn-App if you were tested positive for the virus?**<br><br>1 Definitely enter the result<br>2 Probably enter the result<br>3 Maybe enter the result, maybe not enter the result<br>4 Probably not enter the result<br>5 Definitely not enter the result

Table B2 Question and answer texts of STAI-SKD scale (unofficial translation)

German	English
Im Folgenden finden Sie eine Reihe von Aussagen, mit denen Menschen sich selbst beschreiben. Bitte geben Sie an, wie sehr die jeweilige Aussage angibt, wie Sie sich jetzt in diesem Moment fühlen. Es gibt keine richtigen oder falschen Antworten.<br><br>Überlegen Sie bitte nicht lange und denken Sie daran, diejenige Antwort auszuwählen, die Ihren augenblicklichen Gefühlszustand am besten beschreibt.<br><br>**Ich bin angespannt.**<br><br>1 überhaupt nicht<br>2 ein wenig<br>3 ziemlich<br>4 sehr	In the following you will find a number of statements people use to describe themselves. Please indicate how much each statement reflects how you feel right now. There are no right or wrong answers.<br><br>Please do not think too long and remember to choose the answer that best describes your current emotional state.<br><br>**I am tense.**<br><br>1 not at all<br>2 somewhat<br>3 moderately so<br>4 very much
**Ich bin aufgeregt.**<br><br>1 überhaupt nicht<br>2 ein wenig<br>3 ziemlich<br>4 sehr	**I am agitated.**<br><br>1 not at all<br>2 somewhat<br>3 moderately so<br>4 very much
**Ich bin besorgt, dass etwas schiefgehen könnte.**<br><br>1 überhaupt nicht<br>2 ein wenig<br>3 ziemlich	**I am worried that something could go wrong.**<br><br>1 not at all<br>2 somewhat<br>3 moderately so

4 sehr	4 very much
**Ich bin beunruhigt.**<br><br>1 überhaupt nicht<br>2 ein wenig<br>3 ziemlich<br>4 sehr	**I am disturbed**<br><br>1 not at all<br>2 somewhat<br>3 moderately so<br>4 very much
**Ich bin nervös.**<br><br>1 überhaupt nicht<br>2 ein wenig<br>3 ziemlich<br>4 sehr	**I am nervous.**<br><br>1 not at all<br>2 somewhat<br>3 moderately so<br>4 very much

Table B3 Question and answer texts of COVID-19 political measures support scale

German	English
In Deutschland werden und wurden zur Eindämmung der Corona-Pandemie verschiedene Maßnahmen diskutiert und ergriffen. Wir möchten nun von Ihnen wissen, was Sie von bereits beschlossenen Maßnahmen als auch von möglichen zukünftigen Maßnahmen halten.<br><br>**Welche der folgenden Maßnahmen halten Sie in der heutigen Situation für angemessen?**<br><br>*Bitte geben Sie alle Maßnahmen an, die Sie für angemessen halten.*<br><br>[ ] Schließung öffentlicher Einrichtungen (z.B. Universitäten, Schulen und Kindergärten)<br>[ ] Schließung der Landesgrenzen für Reisende<br>[ ] Verbot von Veranstaltungen mit mehr als 100 Teilnehmern<br>[ ] Allgemeine Ausgangssperre<br>[ ] Einstellung des Nah- und Fernverkehrs<br>[ ] Ich halte keine dieser Maßnahmen in der heutigen Situation für angemessen.	In Germany several measures to contain the COVID-19 pandemic have been discussed and taken. We would now like to know your views on measures already adopted and on possible future measures.<br><br>**Which of the following measures do you think are appropriate in the current situation?**<br><br>*Please name all measures that you think are appropriate.*<br><br>[ ] Closure of public institutions (e.g., universities, schools, kindergartens)<br>[ ] Closure of country boarders for travelers<br>[ ] Prohibition of events with more than 100 participants<br>[ ] General curfew<br>[ ] Suspension of local and long distance public transport<br>[ ] I do not think any of these measures is appropriate in the current situation.
